# Internalizing Symptoms and Their Impact on Patient-Reported Health-Related Quality of Life and Fatigue among Patients with Craniopharyngioma During Proton Radiation Therapy

**DOI:** 10.3390/children11101159

**Published:** 2024-09-25

**Authors:** Belinda N. Mandrell, Yian Guo, Yimei Li, Donna Hancock, Mary Caples, Jason M. Ashford, Thomas E. Merchant, Heather M. Conklin, Valerie Mc. Crabtree

**Affiliations:** 1 Department of Pediatric Medicine and Division of Nursing Research, St. Jude Children’s Research Hospital, Memphis, TN 38501, USA; donna.hancock@stjude.org (D.H.); mary.caples@stjude.org (M.C.); 2 Statistician Sutter Health Care, Palo Alto, CA 94301, USA; yian.guo@sutterhealth.org; 3 Department of Biostatistics, St. Jude Children’s Research Hospital, Memphis, TN 38501, USA; yimei.li@stjude.org; 4 Department of Psychology, St. Jude Children’s Research Hospital, Memphis, TN 38501, USA; jason.ashford@stjude.org (J.M.A.); heather.conklin@stjude.org (H.M.C.); valerie.crabtree@stjude.org (V.M.C.); 5 Department of Radiation Oncology, St. Jude Children’s Research Hospital, Memphis, TN 38501, USA; thomas.merchant@stjude.org

**Keywords:** craniopharyngioma, proton therapy, internalizing behaviors, symptoms, latent class

## Abstract

Objective: The aim of this study was to describe fatigue, health-related quality of life (HRQOL) and brain tumor-associated symptoms after surgical resection and during proton radiotherapy, using latent class analysis (LCA), and to determine if there is class membership change among pediatric patients with craniopharyngioma. Methods: For all patients (*n* = 92), demographic and disease-related/clinical variables were attained, and patient reported outcomes were collected prior to proton therapy, at week three, and at the completion of proton therapy. The mean scores for fatigue, HRQOL, and brain tumor symptoms were compared over time and profiles were identified. Factors that influenced profile status and transition probability were examined. Results: Fatigue, HRQOL, and brain tumor symptoms improved over time during proton therapy; however, a subset remained in the lower profile, profile 1, associated with increased internalizing behaviors, compared to profile 2. Conclusions: Future study should explore the bidirectional relationship of sleep, worry and anxiety in the context of ongoing radiotherapy.

## 1. Introduction

Craniopharyngioma is an uncommon low-grade tumor of the sellar/parasellar region of the brain, with potential invasion into the hypothalamus, pituitary gland, and optic nerve [1]. The treatment goal is to preserve function and reduce treatment-related impairment, through surgery alone or limited surgery and radiotherapy. Although overall survival rates are high (87–95%), visual field disruption, endocrine deficiencies, hypothalamic obesity, sleep–wake disturbance, and neurocognitive impairment are common [2,3]. Long-term health-related quality of life (HRQOL) is significantly impacted due to these impairments resulting from tumor location and treatment of craniopharyngioma.

We recently described the prevalence and clinical predictors of sleep disturbances after surgical resection and prior to radiotherapy in children with craniopharyngioma [4]. Predictors included higher body mass index (BMI) and hypothalamic involvement (HI) at diagnosis. Self-reported HRQOL and brain tumor-specific symptoms were not significantly different between those with hypersomnia, narcolepsy, or absence of sleep–wake disturbance.

Alterations of the hypothalamus secondary to tumor and surgical resection have been well described as contributing to obesity, impacting HRQOL. Beyond obesity and sleep, HI has been associated with a significant impact on behavioral, social and emotional functioning [5,6]. These social and emotional consequences may include anxiety, unkemptness, food preoccupation, anger, and difficulty with peer relationships [7]. Klages et al. [8] recently described an association of HI with greater BMI and sleepiness, and greater fatigue associated with more psychosocial problems. Fragmented sleep and fatigue indirectly predicted poorer HRQOL through psychosocial problems.

Although disease-related variables at the time of diagnosis are important in understanding HRQOL among patients with craniopharyngioma, treatment variables including type of radiation therapy should also be considered. Eveslage et al. [9] recently described the HRQOL of children and adolescents with craniopharyngioma enrolled in the KRANIOPHARYNGEOM 2007 study, a prospective multicenter study with a randomization arm intended to determine the timing of postoperative radiotherapy. Self-assessed HRQOL was compared from baseline to three years after diagnosis. Patients who underwent radiotherapy shortly after resection had a marginally lower HRQOL when compared to those who underwent radiotherapy at the time of progression. Scores were not statistically different when comparing scores among those treated with photon verses proton-beam therapy.

Patient-reported outcomes are essential in pediatric clinical care [10]. Pediatric patients often under report symptoms and perceive symptoms as inherent in treatment [11,12]. Numerous factors including time have been found to influence symptoms and HRQOL, with reported improved HRQOL as time progressed during therapy [13]. However, most HRQOL studies only measure the mean scores over time and fail to identify individual profiles that may be conducive to interventional care strategies [14]. The aim of this study was to describe fatigue, HRQOL and brain tumor-associated symptoms after surgical resection and during proton radiotherapy, using latent class analysis (LCA), and to determine if there is a class membership change among patients over time. The use of LCA provides improved understanding of the interindividual variability of HRQOL and associated demographic and clinical variables during proton therapy. Clinical variables included objective assessment of excessive daytime sleepiness (hypersomnia/narcolepsy), HI grade, BMI, diabetes insipidus (DI), visual acuity, actigraphy-defined sleep onset latency (SOL), wake after sleep onset (WASO), SOL and WASO variability, psychosocial issues (including internalizing problems, anxiety, and depression), and parental socioeconomic status. It was hypothesized that a greater HI would be associated with hypersomnia/narcolepsy, psychosocial issues, and a poorer HRQOL.

## 2. Materials and Methods

### 2.1. Participants

From August 2011 to May 2016, children and adolescents (*n* = 92) diagnosed with craniopharyngioma, self-reported their HRQOL, as well as physical and psychological symptoms, at baseline and weekly during proton therapy. As previously reported, all patients underwent an overnight polysomnography and a next-day multiple sleep latency test (MSLT) [4] and wore wrist actigraphy for 3–5 days. The diagnosis of craniopharyngioma was based on neuroimaging, intraoperative findings (cyst fluid) or histopathology. For those with histopathology, all cases were confirmed as adamantinomatous craniopharyngioma.

### 2.2. Study Design

This single institution longitudinal study enrolled all eligible patients after surgical resection and prior to proton radiotherapy. The total prescribed dose was 54CGE administered at 1.8 CGE per fraction. The time course of administration delivered one fraction per day, 5 days per week, for a period of 6 weeks. The study was approved by the Institutional Review Board. Consent was obtained from all patients aged 18 years and older or parents of patients less than 18 years. Assent was obtained from children according to institutional policy. Demographic, disease-related and clinical variables were collected at baseline, and HRQOL and symptom questionnaires collected during proton therapy at baseline (T1), week 3 (T4) and completion (T7) were used for this analysis.

### 2.3. Demographic and Clinical Variables

Demographic variables included age, sex, race, socioeconomic status (SES), and BMI z score. The disease-related variables were defined by the study team for consistency. Preoperative hypothalamic involvement (HI) in pre-surgical neuroimaging was categorized as grade 0 (no HI), grade 1 (anterior HI), or grade 2 (anterior and posterior HI, including the mammillary bodies) [1,15]. Diabetes insipidus was categorized as present or not, based on prescribed desmopressin. Visual acuity was categorized as no deficits, impaired vision, and unilateral/bilateral blindness. Clinical sleep variables included sleep diagnosis (hypersomnia or narcolepsy), SOL, and WASO, and psychosocial variables included internalizing problems, anxiety, and depression.

### 2.4. Multiple Sleep Latency Test and Actigraphy

The MSLT is an in-laboratory assessment of a patient’s tendency to fall asleep under standardized conditions and was performed after nocturnal PSG in subjects of 6 years and older. The MSLT was performed according to AASM guidelines [16]. The mean sleep latency (MSL) was calculated as the arithmetic mean of all nap opportunities. The number of naps and the corresponding sleep stages were recorded. Criteria for the identification of hypersomnia were according to Tanner staging (prepubescent versus pubescent) and defined as an MSL of  ≤15 min in prepubescent children with Tanner stage 1 and of  ≤10 min in pubescent youth with Tanner stage 2 or greater [17] (1. The MSLT is considered the “gold standard” for objective characterization of daytime sleepiness, but normative data from typically developing children and uniformly accepted cut-off values for pathological sleepiness in children are lacking. We chose a cut-off value for mean sleep latency of <15 min in prepubescent children and <10 min in pubescent youth. This is a deviation from the ICSD-3 recommendation (cut-off value of <8 min), which refers to adults). Tanner staging was documented by the study endocrinologist. Criteria for narcolepsy included an MSL of  ≤15 min in prepubescent children with Tanner stage 1 and of  ≤10 min in pubescent youths with Tanner stage 2 or higher, and a presence of two or more sleep-onset REM periods (2. Refer 1).

The MicroMini Sleep Watch (Ambulatory Monitoring Inc., Ardsley, NY, USA) was used to collect SOL and WASO. This actigraph is a wrist-worn device that contains a biaxial piezoelectric sensor and a microprocessor with programmable epoch length. The algorithm by Sadeh was utilized to compute sleep characteristics including SOL and WASO. Sadeh’s algorithm has been validated against pediatric PSG results [18]. Patients had three nights of data for evaluation.

### 2.5. Health-Related Quality of Life, Fatigue, and Socioeconomic Status

HRQOL was assessed in children and adolescents using the Pediatric Quality of Life Inventory (PedsQL V.4), a 23-item Likert scale measuring the domains of physical, emotional, social, and school function. The scale is internally consistent and clinically valid [19]. The scale scores are reverse scored, transforming the 0–4 scare to 0–100-point scale, with higher scores reflecting a higher HRQOL. The Pediatric Quality of Life Inventory brain tumor module (PedsQL V.3) is a 24-item Likert scale measuring brain tumor-related symptoms in children and adolescents receiving treatment for a brain tumor. The scale measures symptoms of cognitive function, pain and hurt, movement and balance, procedural anxiety, nausea, and worry, with higher scores reflecting a higher HRQOL. The PedsQL brain tumor module is internally consistent [20]. The Peds QL multidimensional fatigue scale (MDFS) evaluates physical, cognitive, and sleep-related fatigue. The 18-item Likert scale is internally consistent and clinically valid in children with cancer and other chronic illnesses, with higher scores representing less fatigue [19]. All scales were completed by children and adolescents aged ≥5 years.

Socioeconomic status (SES) was obtained from the Barratt Simplified Measure of Social Status [21] which utilizes the parental education and occupation data for a composite score. Scores range from 8–66, with higher scores being consistent with higher SES.

### 2.6. Psychosocial Problems

Psychosocial problems were parent-reported through completion of the Behavior Assessment System for Children, Second Edition (BASC-2) [22]. The BASC-2 assesses behavioral, emotional, and adaptive functioning both inside and outside the home. This current study utilized the score for internalizing problems, which is a composite score of the anxiety, depression, and somatization subscales. Normal scores are below 60, scores between 60 and 69 indicate “at risk” behavior, and scores above 70 indicate “significant risk”. The BASC-2 is internally consistent and has good test–retest reliability.

### 2.7. Statistical Analysis

A Wilcoxon signed rank test (two-sided) was used to compare MDFS, Peds QL V.4, and PedsQL V.3 between time points. Latent profile analysis (LPA) and latent transition analysis (LTA) were conducted to identify profiles of patients receiving proton therapy for craniopharyngioma and determine if there was a change in profile membership over time, using MDFS, Peds QL V.4, and PedsQL V.3, at T1 (baseline), T4 (week 3 of proton therapy) and T7 (end of proton therapy). The LTA then identified profiles at each time point simultaneously. The optimal number of profiles were identified at each time point, as well as the transition of profiles over time. Factors that affected profile status and transition probability were also studied.

For LPA, variances and covariances of the Peds QL V.4, PedsQL V.3, and MDFS could be freely estimated across profiles. The number of profiles was determined based on fit of data and classification quality as well as interpretability of the profiles. Classification quality was evaluated using the entropy statistic. Fitting criteria included the Akaike information criterion (AIC), Bayesian information criterion (BIC) and the Bootstrapped Likelihood Ratio Test (BLRT) comparing the k-profile model to the k-1-profile model. Once the optimal number of latent profiles was identified, subjects were classified into the latent profile with the highest estimated posterior probability.

For LTA, a subject’s latent profile status could change over time. To ensure that the latent profiles estimated by the model are consistent across time, means, variances and covariances of the three measures in the same profile were restricted to be equal across all three time points. The number of latent profiles and the profile status of subjects were determined similarly as in LPA. Then, the profile status at the three time points and the transition probability across time were summarized and compared, and their relationship to demographic and clinical variables was analyzed using logistic regression. Statistical analyses were conducted using Mplus Version 8.2 and R version 4.0.2.

## 3. Results

The patient demographic and clinical characteristics are described in Table 1. The mean age was 10.5 years, 51% were female, 64% were white, and the average BMI z-score was 1.34. Most had grade 2 HI, were receiving treatment for diabetes insipidus, and had a diagnosed sleep disorder of hypersomnolence. The descriptive statistics of MDFS (fatigue), Peds QL V.4 (global HRQOL), and Peds QL V.3 (brain tumor symptoms), at T1, T4 and T7 are presented in Table 2, with overall means and medians less than 80 and a wide range in scores. Table 3 depicts the change in scores over time. Although the global scores are less than 80, over time, patients self-reported a significant improvement in fatigue (*p* < 0.01), HRQOL (*p* = 0.04) and brain tumor symptoms (*p* < 0.01) from T1 to T4; however, patients did not report significantly improved fatigue, HRQOL or brain tumor symptoms from T4 to T7. Fatigue (*p* = 0.03) and brain tumor symptoms (*p* = 0.01) significantly improved from T1 to T7, without a significant change in patient-reported HRQOL.

The means and standard deviations of fatigue, global HRQOL, and brain tumor symptoms were estimated using the LTA model. Mean scores were calculated and plotted by time and profile from the original data after patients were assigned profile memberships at each time point. Profile 1 has the lowest mean scores across all time points, with profile 3 having the highest mean scores across all time points.

**Figure 1:**
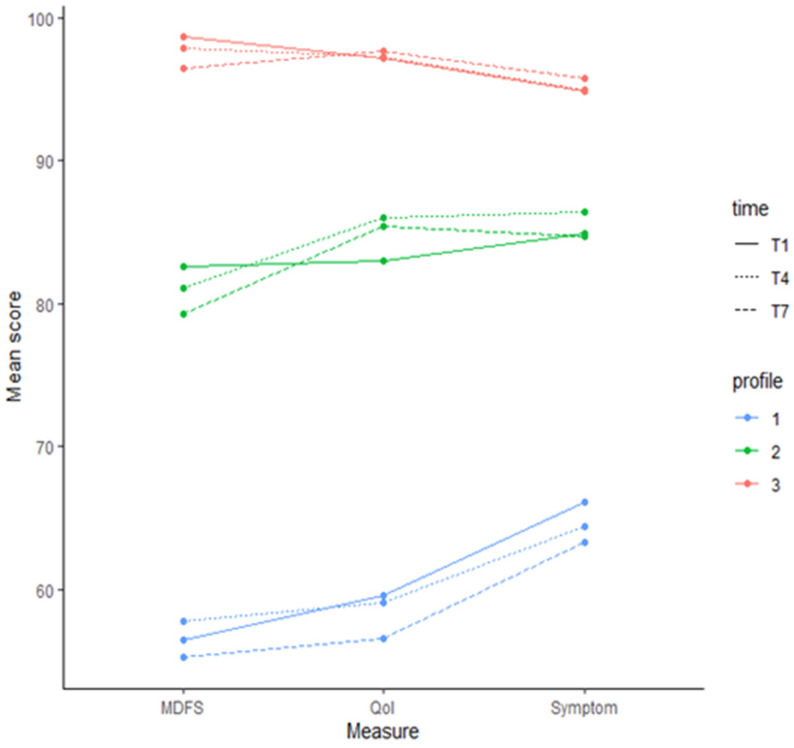

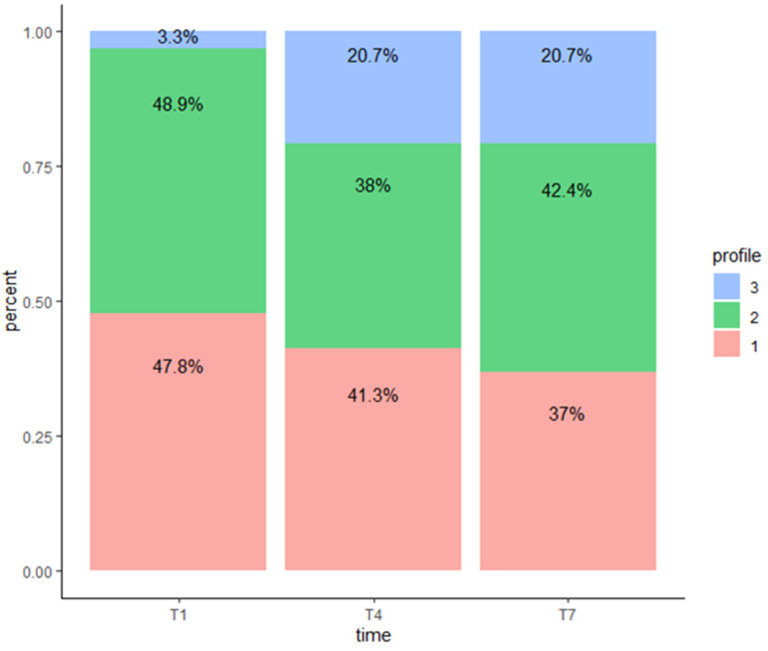
Latent profiles by time point estimated by LTA model.

From T1 to T4, the number of patients in profile 1 decreased from 44 (47.8%) to 38 (41.3%), with nine patients moving to profile 2 and three patients moving from profile 2 to profile 1. In profile 2, the number of patients decreased from 45 (48.9%) to 35 (38%), and 16 patients moved from profile 2 to profile 3. Patients in profile 3 increased from three (3.3%) to nineteen (20.7%). From T4 to T7, the number of patients in profile 1 decreased from thirty-eight (41.3%) to 34 (37%), with four patients moving to profile 2. The number of patients in profile 2 increased from 35 (38%) to 39 (42.4%). One patient in profile 2 moved to profile 3 and another in profile 3 moved to 2. There were 34 patients who remained in profile 1 throughout the course of proton therapy. Assessing the profile transition across time, we found that most patients either remained in the same profile or moved to a superior profile over time (Figure 1).

### Association between Covariates and Profile Status

It is important to understand the clinical characteristics of patients who remained in profile 1. Therefore, we performed logistic regression models for the patients in profile 1 compared to those in profile 2 and for the patients in profile 1 compared to those in profile 3. Thirty-four (37%) patients remained in profile 1 throughout the course of proton therapy, and fifty-three (58.7%) remained in a higher profile or transitioned to a higher profile during therapy.

To evaluate the factors associated with the profile status at each time point, we treated profile status as a categorical outcome and performed univariate logistic regression on baseline age, sex, BMI z-score, DI, excessive daytime sleepiness (yes/no), and HI grade (0–1 vs. 2), average SOL, SOL variability as defined by the standard deviation of SOL, average WASO, WASO variability as defined by standard deviation of WASO, internalizing symptoms, and SES at T1, T4 and T7. Comparing profile 2 classification to profile 1 at T1, the BMI z-score was significantly (*p* = 0.03) associated with the probability of being in profile 2 vs. profile 1, with a one-unit increase in BMI z-score, and the probability of being in profile 2 decreased by 39%. At T4 and T7, hypersomnia had trending significance (*p* = 0.07; 0.08) and its presence increased the probability of profile 1. Parent-reported internalizing problems (T1 *p* = 0.02; T4 *p* = 0.00, T7 *p* = 0.01) were statistically associated with the probability of profile 1.

The multivariate multinomial regression model included variables of significance (*p* < 0.05) and trending significance (*p* < 0.1) and included BMI z-score, hypersomnia, and internalizing problems, as shown in Table 4. Comparing profile 2 and profile 1, internalizing problems were associated with profile 1 (T1 *p* = 0.05; T4 *p* = 0.01; T7 *p* = 0.02) across all time points. Variables of BMI z-score, hypersomnia, and internalizing problems were not significant between those in profile 3 and profile 1.

## 4. Discussion

The descriptive analysis found that patients undergoing proton therapy for treatment of craniopharyngioma reported a moderate improvement in HRQOL, brain tumor-related symptoms and fatigue over time. However, we identified three latent symptom profiles at each time point that identified patients at risk of poorer self-reported HRQOL. Profile 1 was classified as reporting poorer, more and higher fatigue, profile 2 was classified as reporting moderate HRQOL, brain tumor-related symptoms and fatigue, and profile 3 was classified as reporting a high HRQOL and fewer brain tumor-related symptoms or fatigue. Within these profiles, we found that patients in profile1 at T1 were more likely to remain in this profile during proton therapy.

Surprisingly, we did not find an association between profile classification and disorders of hypersomnolence or HI. This could be representative of most patients presenting with hypersomnia/narcolepsy (75%) and grade 2 HI (60.2%), thus limiting variability in detecting associations. As this is a sleepy cohort, nighttime sleep does not appear to drive daytime symptoms during proton therapy. Klages et al. [8] described similar findings with no indirect effects of HI on psychosocial behaviors and HRQOL through BMI, fragmented sleep, or daytime sleepiness. The study described a significant direct effect between fatigue, HRQOL, and psychosocial problems. Fatigue is recognized as a significant predictive factor for poorer HRQOL among those who were likely to remain in this profile throughout the proton treatment trajectory. Given these findings, it is difficult to ascertain whether patients with more internalizing symptoms are more likely to be hypervigilant to other symptoms, thereby leading them to fall into the profile in which they describe worse HRQOL, brain tumor symptoms, and fatigue or if patients with worse daytime symptoms are more likely to experience internalizing symptoms. Likely, this reflects a self-perpetuating cycle in which daytime brain tumor-related symptoms and fatigue drive poorer HRQOL, all of which contribute to increased internalizing symptoms, which in turn exacerbate daytime fatigue and brain tumor-related symptoms. These clusters of symptoms point to the importance of multifactorial interventions to address a multitude of challenges experienced by pediatric patients with craniopharyngioma.

Limitations to this study include a small sample size across each profile that limits our power in determining differences between those in profile 1 and profile 3. Although our sample size is small, craniopharyngioma is a rare tumor, and this is a sizable representation. This limitation will be addressed with continued study of newly diagnosed patients, with the use of standardized patient-reported batteries across trials.

## 5. Conclusions

Patients treated for craniopharyngioma should be frequently assessed for psychosocial impairments. If present, psychosocial and/or pharmacologic interventions should be initiated. Further study that explores profile class across the trajectory of survivorship is warranted to determine if internalizing problems continue to co-exist with symptoms of fatigue and poorer HRQOL beyond the end of treatment. Survivors of craniopharyngioma are known to be at significant risk for HI-associated obesity and sleep disturbance. Therefore, further study may find that these variables are associated with fatigue and HRQOL over time. Numerous intervention studies are warranted for this patient population in addition to psychosocial interventions. These interventions should address weight management, sleep health (including daytime sleepiness and obstructive sleep apnea), physical activity, and social skills training with increased opportunities for socialization. Additionally, those with sleep variability may engage in cognitive behaviors that impede sleep including worry and anxiety [23]. Such interventions should include instruction in healthy sleep practices with consistent times to bed and rise, stimulus control (e.g., no electronic or phone use in bed), time in bed for only sleep, and relaxation techniques. Future studies should explore the bidirectional relationship between sleep, worry, and anxiety in the context of survivorship.

## Figures and Tables

**Table 1:** Demographic and clinical characteristics (*n* = 92).

Characteristic	*n* (%)
Age (Years)	10.5 (4.0) *
Sex	
Female	47 (51.1%)
Male	45(48.9%)
Race	
White	59 (64.1%)
Black	16 (17.4%)
Asian	3 (3.3%)
Multiple	9 (9.8%)
Other	3 (3.3%)
Unknown	2 (2.2%)
BMI z-score	1.34 (1.03)
Hypothalamic involvement	
Grade 0/1	37 (40.2%)
Grade 2	55 (59.8%)
Diabetes Insipidus	
No	39 (42.4%)
Yes	53 (57.6%)
Narcolepsy	
No	53 (57.6%)
Yes	30 (32.6%)
Unknown	9 (9.8%)
Hypersomnia	
No	44 (47.8%)
Yes	39 (42.4%)
Unknown	9 (9.8%)
Positive Sleep Diagnosis	
No	14 (15.2%)
Yes	69 (75.0%)

* Mean age (SD).

**Table 2:** Descriptive statistics of MDFS, global HRQOL, and brain tumor symptoms.

Measure	N	Mean (SD)	Median (Range)
T1 *			
MDFS	92	71 (19)	71 (3–100)
HRQOL	92	72 (16)	72 (25–100)
Symptoms	92	76 (13)	76 (39–96)
T4 *			
MDFS	80	75 (20)	75 (26–100)
HRQOL	80	77 (19)	77 (26–100)
Symptoms	79	79 (15)	79 (28–100)
T7 *			
MDFS	76	74 (21)	74 (7–100)
HRQOL	76	77 (20)	77 (14–100)
Symptoms	75	79 (16)	79 (28–100)

* T1—baseline; T4—week 3 of proton therapy; T7—completion of proton therapy.

**Table 3:** Change in MDFS, global HRQOL, and brain tumor symptom score between time points (median, range and *p*-values).

Measure	Change between T1 *, T4 *	p_T1 *, T4 *_	Change between T4 *, T7 *	p_T4 *, T7 *_	Change between T1 *, T7 *	p_T1 *, T7 *_
MDFS	6 (−31, 42)	<0.01	−2 (−44, 39)	0.55	6 (−29, 53)	0.03
HRQOl	3 (−26, 33)	0.04	0 (−14, 26)	0.86	3 (−31, 39)	0.07
Symptom	7 (−33, 38)	<0.01	0 (−24, 28)	0.92	7 (−27, 40)	<0.01

* T1—baseline; T4—week 3 of proton therapy; T7—completion of proton therapy.

**Table 4:** Multivariate multinomial logistic regression of profile status on BMI, hypersomnia and internalizing problems.

Profile 2 vs. Profile 1						
	T1 *		T4 *		T7 *	
Variable	RR (95% CI)	*p*	RR (95% CI)	*p*	RR (95% CI)	*p*
BMI z-score	0.65 (0.37, 1.15)	0.14	0.95 (0.5, 1.8)	0.87	1.11 (0.59, 2.06)	0.75
Hypersomnia	1.26 (0.45, 3.47)	0.66	0.49 (0.15, 1.55)	0.22	0.56 (0.18, 1.72)	0.31
Internalizing prob.	0.95 (0.91, 1)	**0.05**	0.93 (0.88, 0.98)	**0.01**	0.94 (0.89, 0.99)	**0.02**
**Profile 3 vs. profile 1**						
Variable	RR (95% CI)	*p*	RR (95% CI)	*p*	RR (95% CI)	*p*
BMI z-score	1.6 (0.24, 10.66)	0.63	0.62 (0.31, 1.26)	0.19	0.71 (0.35, 1.45)	0.35
Hypersomnia	0.97 (0.05, 18.56)	0.98	0.8 (0.21, 3.02)	0.74	0.58 (0.15, 2.21)	0.42
Internalizing prob.	0.99 (0.88, 1.1)	0.82	0.96 (0.91, 1.02)	0.19	0.96 (0.9, 1.02)	0.15

* T1—baseline; T4—week 3 of proton therapy; T7—completion of proton therapy.

## Data Availability

Data are unavailable due to privacy and ethical restrictions.
